# Atopic Dermatitis in Latin America: Considerations on Epidemiology, Clinical and Laboratory Features, Ethnic/Racial Variations, and Therapeutic Management

**DOI:** 10.3390/jcm12103419

**Published:** 2023-05-11

**Authors:** Georgia Biazus Soares, Raquel Leao Orfali, Beatriz Lacerda Averbach, Gil Yosipovitch, Valeria Aoki

**Affiliations:** 1 Dr. Phillip Frost Department of Dermatology and Cutaneous Surgery, Miami Itch Center, University of Miami Miller School of Medicine, Miami, FL 33130, USA; gbs52@med.miami.edu (G.B.S.); yosipog@gmail.com (G.Y.); 2 Hospital das Clinicas HCFMUSP, Faculdade de Medicina, Universidade de Sao Paulo, Sao Paulo 05403-000, Brazil; beatrizaverbach@gmail.com (B.L.A.); valeria.aoki@gmail.com (V.A.)

**Keywords:** atopic dermatitis, Latin America, epidemiology, ethnic/racial, clinical features

## Abstract

Latin America (LA) encompasses about 8.5% of the world’s population, exhibits ethnic/racial heterogeneity and social inequality. We hereby present a 20-year literature review (2004–2023) on epidemiology, diagnosis, clinical and laboratory features, quality of life and management of atopic dermatitis (AD) in LA. Highest AD prevalence for children aged 6–7 years was reported in Ecuador (22.5%) and Colombia (20.9%), for adolescents in Colombia (24.6%) and for all ages, in Brazil (20.1%). Regions with a predominantly Black population in LA varied significantly, ranging from 4.4% in Northern Brazil to 10.1% in Cuba, indicating genetic variation among African subgroups. Filaggrin loss-of-function mutations showed variants seen in Europeans in 9.3% of Chilean patients and studies in Brazil revealed impaired expression of filaggrin and claudin-1 in the skin but increased expression in conjunctival epithelia of AD patients. The most reported AD features included erythema, pruritus, and dry skin, with marked lichenification. Severe pruritus was reported by 54.4% of patients and a high impact on quality of life was detected in 50% of adults with AD. In Brazilian referral hospitals, 65.6% of patients were classified as having severe AD, and 56% had one or more hospitalizations during their lifetime, indicating a need for better disease control. Diagnosing AD is challenging due to broad clinical features, ethnoracial variations and lack of universal diagnostic criteria. Furthermore, lack of physician training, barriers to medication access, and socioeconomic inequalities hinder effective disease management in LA.

## 1. Introduction

Atopic dermatitis (AD) is a chronic, relapsing inflammatory skin disease that often presents in childhood and is estimated to affect 15–20% of children and 1–3% of adults worldwide [1]. The pathophysiology of AD is multifactorial, and clinical characteristics are varied. AD is associated with a high disease burden that has a profound impact on quality of life [2,3]. Latin America (LA) encompasses about 8.5% of the world’s population and is one of the regions with the most social inequality [4]. This inequality, combined with diverse racial/ethnic, geographical, and social elements in the region contributes to healthcare disparities that make understanding and treating conditions such as AD challenging. The objective of our study is to fill this gap by applying a systematic approach to screening the literature specifically for studies examining the epidemiology, clinical and laboratory features, ethnic/racial variations, and therapeutic management linked to AD in Latin America. The authors describe future directions that may help alleviate present challenges.

## 2. Materials and Methods

### Review Strategy

PubMed, Embase and Google scholar databases were the databases utilized for this review, with the search terms: (“atopic dermatitis” OR “atopic eczema”) AND (“Latin America”). The studies were then assessed based on title and abstract for relevance to AD and association with prevalence and ethnicities. We included English-language articles published between 2004 to 2023. The considered relevant papers were accessed and included in the review.

## 3. Results

**Figure 1:**
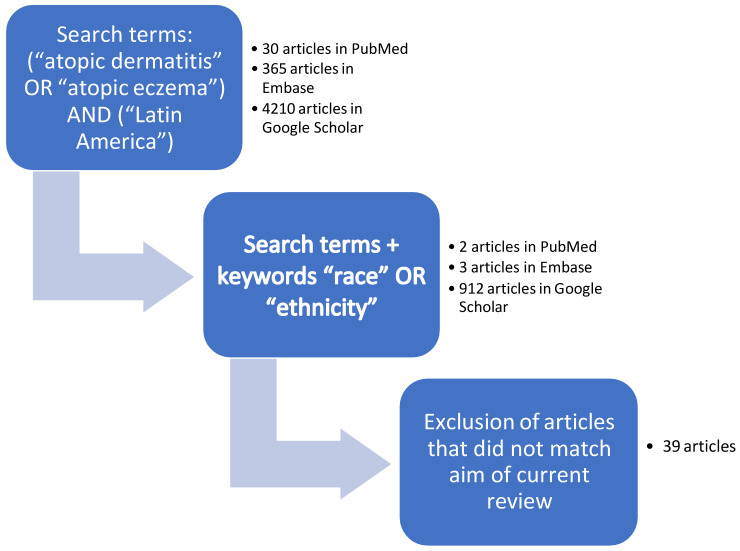
Results of the web search. Graphic illustration of the web search, keywords included in the main search and selection of articles.

The search terms resulted 30 articles in PubMed, 365 in Embase, and 4210 in Google Scholar. When including the keywords (“race” OR “ethnicity”) in the main search, the results showed only 2 publications in PubMed, 3 in Embase, and 912 in Google Scholar, but still with subjects that did not match with the purpose of this article. We excluded all articles that did not match the aim of this current review and accessed the relevant papers related to the theme. Figure 1 illustrates the results of the search.

### 3.1. Epidemiology of AD

Two large multicenter studies have provided important data on the prevalence of AD in Latin America. The International Study of Asthma and Allergies in Childhood (ISAAC) Phase 1 showed that the prevalence varied in different Latin American countries, ranging from around 4% in Mexico to as high as 10.9% in Chile in 6 to 7-year-olds and 10.8% in Paraguay in 13 to 14-year-olds [5]. Phase 3 of ISAAC was completed seven years later and included significantly more centers than Phase 1, spanning 14 Latin American countries. For children ages 6 to 7, the Latin American centers with the highest AD prevalence included Quito, Ecuador (22.5%) and Barranquilla, Colombia (20.9%). For adolescents, prevalence ranged from 2.8% in the Mexicali Valley, Mexico to 24.6% in Barranquilla, Colombia [5]. Another international, multicenter study found that Brazil had the highest prevalence of AD in all age groups among Latin American countries (20.1%) [6]. Smaller studies have also been conducted to examine the prevalence of AD within Latin American countries. For example, Garcia et al. reported that in Bogota, Colombia, 42.3% of children in their study had dermatological conditions, and 6.5% had AD [7]. Another study in Brazil found mean AD prevalence rates of 8.2% in schoolchildren and 5.0% among adolescents [8]. A recent study evaluating one-year prevalence of AD in Brazil through a population-based telephone survey, revealed that the age-adjusted prevalence of AD was 2.27% [9]. Data on the epidemiology of adult AD in Latin America are extremely limited, with one multicenter retrospective study in Brazil named ADAPT SA, a multicentric, non-interventional study to describe clinical features and disease management of adult patients with atopic dermatitis followed at tertiary hospitals [10].

Studies in the US and Europe have shown a higher AD prevalence in Black children, but the mean prevalence in LA regions with a predominantly Black population varied significantly, ranging from 4.4% in Northern Brazil to 10.1% in Cuba [5,11]. This supports the notion that there is genetic variation among African subgroups. Furthermore, certain genes involved in immune regulation and epithelial barrier function are involved in the pathogenesis of AD in specific ethnic groups. Filaggrin loss-of-function mutations, for example, play a key role in the development of AD in European populations [11]. The population in some of the LA countries is predominantly of European origin. A study in Chile showed that filaggrin variants commonly seen in European patients with AD were observed in 9.3% of Chilean patients [12]. There was an impaired expression of filaggrin and claudin-1 (a tight junction protein), in the skin of Brazilian patients. Interestingly, these proteins were found to be increased in conjunctival epithelial cells of patients with AD when compared to healthy controls, which could reflect a reactive response to AD-induced inflammation [13]. Differences in climate, humidity, and UV exposure can also all contribute to disease prevalence. A positive correlation between prevalence of AD and latitude was found in ISAAC Phase 1, and ISAAC Phase 3 further showed that the prevalence and severity of AD were higher in centers near the Equator [5,14]. The association between latitude and the prevalence of AD symptoms has been further demonstrated in other Latin American studies [8].

### 3.2. Immunological Studies in AD

One Brazilian analysis found increased expression of IL-22 in AD dermal lesions, emphasizing a possible Th22 deviation in these patients [15]. In another study, Orfali et al. reported increased IL-17 in both the serum and skin lesions of AD patients when compared to controls, as well as increased IL-22-expressing CD4/CD8 T cells in AD lesions and an impaired CD4 cytokine response after staphylococcal enterotoxin administration [16].

**Figure 2:**
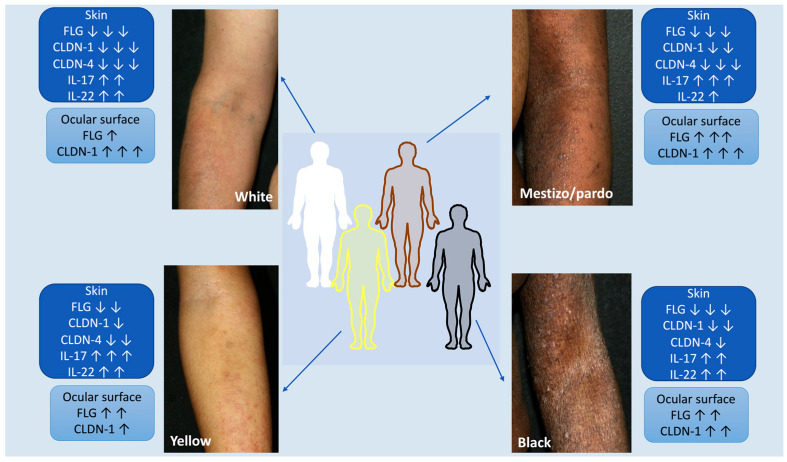
Atopic dermatitis and ethnic/racial variations in a Brazilian pilot ongoing cohort study: clinical presentation, expression of skin barrier proteins in cutaneous and ocular epithelia and in situ immunological profile of Th17 and Th22 axes. The initial findings show differences in the expression of skin barrier proteins present in the cutaneous and ocular epithelia of AD patients, with variations in the ethnoracial profiles (Unpublished data). The figure was partly generated using Servier Medical Art, provided by Servier, licensed under a Creative Commons Attribution 3.0 unported license.

An ongoing pilot study from a Brazilian referral university hospital on atopic dermatitis in adults evaluated the clinical presentation, the expression of skin barrier proteins in cutaneous and ocular epithelia and the in situ immunological profile of Th17 and Th22 axes. The initial findings showed distinct patterns regarding the expression of skin barrier proteins in the cutaneous and ocular epithelia of AD patients, with variations in ethnic/racial profile (Figure 2—Unpublished data).

### 3.3. AD: Diagnosis and Clinical Practice Guidelines

A study in Mexico found that 42% of physicians used these criteria to achieve a diagnosis of AD, whereas a survey completed by AD Therapeutic Area Experts throughout Brazil showed that 82% of AD experts use it [17,18]. However, there are other diagnostic criteria available. Countries in Latin America such as Colombia, Argentina, and Brazil have their own clinical practice guidelines for AD diagnosis and management [19]. Adhering to diagnostic criteria is extremely important to objectively evaluate patients and avoid misdiagnoses. The diagnosis of AD may be delayed in LA due to the presence of other tropical cutaneous diseases that present with pruritus and lichenification such as scabies, papular urticaria, and miliaria. Studies in tropical countries showed that up to 80% of infectious dermatoses such as scabies were initially misdiagnosed as AD [20]. The use of ancillary tests to assist in the diagnosis of AD has also been discussed. AD has been characterized as extrinsic (IgE mediated) and intrinsic (non-allergic), and levels of IgE may correlate with disease severity [21]. Therefore, measuring levels of this immunoglobulin may be helpful when diagnosing AD. In one epidemiologic study of 80 Brazilian patients, the mean circulating IgE level was 18,340 UI/mL [21]. However, this must be interpreted carefully in Latin America, for a large part of the non-allergic population may have increased IgE levels due to the high incidence of helminth infections, which could cause sensitization [22]. A study conducted in a poor urban area of Brazil found positive associations between the number of helminth infections and the production of allergic inflammatory markers including peripheral eosinophilia, increased serum IgE levels, and helminth antigen-stimulated Th2 cytokine production [23].

### 3.4. Clinical Characteristics, Impact on Quality of Life, and AD Assessment

The Brazilian ADAPT study analyzed the clinical characteristics of AD and found that most patients presented with flexural lesions in the popliteal and antecubital regions, and lesions distributed on extensor surfaces such as the extremities and trunk [10]. At the first visit, the main AD reported features were erythema (54.5%), pruritus (55.1%), and dry skin (48.7%), and lesions had the characteristic lichenified or eczematous morphology [10]. In a cohort of 1,650 Argentinian patients with AD, 40% experienced high itch intensity and frequency, and 96% reported bleeding and suppuration [24]. A cross-sectional study in Colombia found that disease distribution was mostly flexural and combined with either eyelid dermatitis, hand eczema, or cheilitis, and that most patients suffered from moderate disease when evaluated using body surface area (BSA) and eczema area and severity index (EASI) score [25]. In a recently published abstract, results from a study evaluating the burden of AD in Brazil, Mexico, and Argentina found that severe pruritus (Worst Pruritus NRS > 7) was reported by 54.4% of patients and with effect on the quality of life close to 50% of the 180 evaluated patients [26].

A Brazilian study evaluated AD patients using the Beck depression inventory, the inventory of stress symptoms for adults, and the dermatology life quality index (DLQI) and found that 38.7% of patients had moderate to severe depressive symptoms and 22.6% had severe depressive symptoms. Furthermore, 73.3% of patients experienced symptoms of psychological stress, and 45.2% reported a significant QoL impairment. Pruritus was one of the main symptoms that contributed to these findings [27]. In Argentina, one survey-based study reported that AD impacted quality of life in 85.6% of participants [24]. Sleep disorders are also a common comorbidity and contribute to reduced QoL, as well as emotional and functional impairment. Latin American children with AD have higher values on the Children’s Sleep Habits Questionnaire (CSHQ) than controls, meaning that they experienced more sleep disorders including sleep anxiety, night awakening, parasomnias, and daytime sleepiness. High CSHQ scores were also found to correlate with increased disease severity [28]. Unfortunately, these psychological factors are not usually adequately addressed in many LA countries, with only 11.8% of AD patients reporting they received therapy or psychological support in the ADAPT study [10].

Clinical assessment of atopic dermatitis utilizes a variety of validated tools. Sanchez et al. found that using international assessment guidelines such as SCOring Atopic Dermatitis (SCORAD) and DLQI significantly reduced the severity of symptoms and improved the quality of life in a cohort of Colombian AD patients [29]. Brazilian studies have shown that 65.6% of patients were classified as having severe disease, and 56% of AD patients had one or more hospitalizations during their lifetime, indicating a need for better disease control [10,21]. Other Latin American studies suggest that comorbidities are linked to increased disease severity, further highlighting the need to accurately assess the disease burden in these patients [19].

### 3.5. Therapeutic Management of AD

First-line therapy in most Latin American clinical practice guidelines consists of emollients, baths, and irritant avoidance [17,19]. Medications such as topical steroids and topical calcineurin inhibitors (TCI) are also used as first- and second-line therapy [17,19,30]. Although topical steroids are considered to be easily accessible, efficacy and costs may vary, and they can be associated with undesirable side effects such as cutaneous atrophy and adrenal suppression [17]. TCIs are recommended for use in more sensitive areas such as the face and genitals, although they are not widely used due to cost [17,30].

Phototherapy, oral corticosteroids, and systemic immunosuppressants such as methotrexate (MTX) and cyclosporine are used for more severe cases that do not respond to topical therapy, but each comes with its unique set of challenges [17]. Although phototherapy is an effective and safe method, it is not widely used for dermatitis in Latin America and is not easily accessible for patients who live far away from phototherapy facilities [22,31]. Oral corticosteroids are associated with significant side effects and are not meant for chronic use, although they are often used in this manner in Latin America [22]. For example, a study in Brazil found that corticosteroids were the most frequently used systemic AD treatment, with 32.6% of patients taking oral steroids for a mean duration of 65.4 days [10]. Most systemic immunosuppressants are not approved for AD treatment in Latin American countries and are used off-label [17]. These therapies also require frequent monitoring and may be costly. Guidelines developed by the Latin American Society of Allergy, Asthma, and Immunology AD Committee provided strong recommendations for the use of cyclosporine A and weak recommendations for the use of methotrexate, stating that further studies are needed to evaluate these treatments in Latin America [22]. However, both therapies are still commonly used, with 24.1% of patients on cyclosporine and 13.4% of participants on MTX in a Brazilian cohort of AD patients [10]. Furthermore, a recent Brazilian study reported significant reduction of EASI, SCORAD, and pruritus after 24 weeks of MTX therapy, thus recommending the use of this drug in refractory moderate to severe AD [32]. Biologic agents such as dupilumab are becoming more widely used in Latin America as countries continue to approve its use, with a few countries such as Brazil even approving it for children ages 6 to 11 years [19]. JAK inhibitors such as upadacitinib and baricitinib have also been approved in various Latin American countries [31]. Unfortunately, accessibility is limited due to cost, public vs. private coverage, and lack of objective measures assessing disease severity (which are required for drug approval) [31]. Furthermore, patients may opt to use alternative or complementary therapies, even though randomized controlled trials are needed to prove their efficacy in AD. One study from Brasilia, Brazil showed that 63.5% of children with AD had used alternative therapies such as homeopathy or phytotherapy to manage their disease [33]. Another Colombian study reported that 37% of patients used alternative medicine as a treatment option [25].

Table 1 summarize the main findings of the articles included in this review.

## 4. Discussion

Latin America comprises a complex geographic region, with marked ethnoracial heterogeneity. LA’s population is known, to date, as the largest admixed population in the globe, due to migration currents from Europe, Africa and Asia over the centuries, generating wide geno/phenotype diversity [34]. The base of official records relies on self-identification, which may differ from the researcher/physician evaluating the individual, or phenotypic classification. Moreover, studies performed in countries in LA show that patients with skin of color are more associated with poorer health [35].

### 4.1. Epidemiology of AD

Limited epidemiology data on the prevalence of AD in LA have been published. This paucity of data has been attributed to several factors, including lack of objective diagnostic criteria and testing, as well as unstandardized nomenclature [17]. The differences in prevalence studies can be partially attributed to the fact that AD is a complex disease that involves an interplay between genetics, immunologic factors, and the environment, all of which vary significantly across different regions of Latin America. The population in this region is comprised of a diverse group of ethnic and racial backgrounds, with ancestry studies showing a high genetic and phenotypic differentiation of Africans, Europeans, and Native Americans, as well as a variable admixture in different regions of Latin America [34].

### 4.2. Immunological Studies in AD

The pathophysiology of AD is known to involve an immune component. The altered immune response is a Th2-driven inflammation with cytokines such as IL-4 and IL-13 playing a key role in promoting inflammation and pruritus. Other pathways with the involvement of a Th1 response and of regulatory T-cells such as Th17 and Th22 in AD pathogenesis have also been recently explored [16,21,36]. Understanding differences in AD immune phenotypes across various regions is crucial to developing and implementing efficacious, targeted treatments for the disease.

Although a Th2 inflammatory response has been reported across various races and ethnicities, a Th1-skewed response is most commonly seen in European Americans when compared to African Americans or patients of Asian ethnicity [40]. Furthermore, Asian patients have been shown to have a significantly higher Th17 and Th22 response in AD skin [41]. There have been few immunological studies performed in Latin America, and the immunologic phenotypes of AD patients in LA, as well as the impact of ethnoracial variations influencing the immune dysfunction is still open to be explored.

### 4.3. AD: Diagnosis and Clinical Practice Guidelines

The diagnosis of AD is primarily based on clinical features. One of the most widely used diagnostic criteria was established by Hanifin and Rajka, and consists of basic characteristics such as pruritus, typical morphology and distribution, a chronic relapsing disease course, and history of atopy, as well as other minor criteria that contribute to the diagnosis [42].

Despite the availability of clinical practice guidelines, diagnosing AD remains a challenge in Latin America. In one Argentinian survey, up to 60% of patients had to visit at least three physicians prior to receiving their AD diagnosis, and 25% of patients reported that their AD diagnosis was delayed by at least 5 years [24]. When analyzing a cohort of patients with AD from Brazil, a country of mixed races, it is noticeable that severity scores for AD, such as EASI [43] and vIGA [44,45] are adequate for those patients where the erythema is visible, including white and mestizo patients, where the erythema is still visible. In white AD individuals, there is even a correlation of severity scores and pruritus, indicating that they may reflect disease activity. This correlation, however, is not seen in the black subjects, where the results show difficulties in adapting the vIgA, and maybe indicating that other skin lesions should be valued, such as lichenification. Moreover, even though the immune dysfunction of AD is Th2-driven, variations in the immune axis, with more, or less involvement of the Th17 or Th22 depending on the ethnic profile of AD patients may impact on therapeutic responses to targeted treatments over time [40,46].

### 4.4. Clinical Characteristics, Impact on Quality of Life, and AD Assessment

The clinical presentation of AD varies amongst different populations. It is important to note that the ADAPT study consisted of a predominantly white patient cohort (71.7%), which is not representative of the diverse ethnic population found in other regions of Brazil. AD patients of African descent are more likely to present with lesions involving the extensor surfaces, and flexural dermatitis is less common [11]. Furthermore, atypical presentations such as perifollicular accentuation of lesions and lichen-planus-like lesions are more commonly observed in darker-skinned individuals [11]. Therefore, physicians should be aware that clinical characteristics of AD may differ in Latin American countries depending on racial and ethnic backgrounds. Interestingly, the race and ethnicity reported in many Latin American studies tends to be based on self-identification, which can differ from examiner-assigned race and ethnicity. For example, studies show that Brazilians tend to self-identify as white more often than interviewers categorized them as white, and Peruvians tend to self-identify as mixed-race more often. This may affect the reliability of the association between race and ethnicity and different aspects of disease [35].

AD is known to significantly impact quality of life (QoL) and has a detrimental effect on mental health, school and work performance, and social interactions. Furthermore, caregivers and families of AD patients can also have their quality of life negatively impacted by the disease. The EPI-CARE study, conducted in multiple countries to assess the impact of pediatric AD on caregivers and family, found that across geographic regions—including Latin America—AD severity correlated with higher scores on the Dermatitis Family Impact questionnaire, indicating a more severe caregiver burden. Parents and caregivers also reported a higher number of missed workdays due to their child’s AD, and this correlated with worsening AD severity [37].

The most commonly clinician-reported assessments to evaluate disease extent, activity, and severity include the SCORAD, the EASI, and the Investigator Global Assessment (IGA). Patient-reported outcomes applied to evaluate patient-perceived symptoms and effects of AD on QoL include the Patient Oriented Eczema Measure (POEM), Atopic Dermatitis Control Tool (ADCT), and patient-oriented SCORAD. The use of these validated instruments is extremely important for quantifying disease burden, treatment response, and patient satisfaction. However, many of these assessments are not used in Latin America due to lack of validation that is needed to account for linguistic and cultural differences [19].

### 4.5. Therapeutic Management of AD

Many factors influence the AD management in the Latin American region. Access to specialized care in many countries is limited due to lack of specialists, high patient volumes, socioeconomic barriers, and lack of AD awareness and training among primary care physicians [32]. The number of dermatologists per 100,000 inhabitants varies depending on the country, ranging from 1.2 in Mexico to 9.2 in Uruguay [31]. Socioeconomic inequities also hinder the access to and availability of certain AD treatments [31].

Despite recent advances in the era of targeted-oriented therapies, many patients in LA are not satisfied with the care they receive for their AD. Results from studies in Argentina and Brazil show that 40.5% of patients were not satisfied with their treatment regimen, and 33.7% of patients discontinued their medication due to poor effectiveness [10,24]. It is important to note that AD often requires a multidisciplinary team for disease assessment and management, emotional support, and treatment of comorbidities, and that this approach can be difficult to accomplish in many Latin American countries [17,19].

## 5. Future Directions

**Figure 3:**
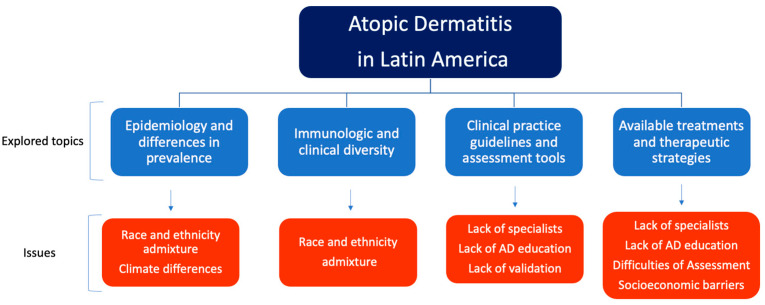
Explored topics and major issues of AD in Latin America. Graphic illustration of the main topics discussed in this review and the gaps that still need to be addressed in Latin America.

There are many knowledge gaps and unmet needs that need to be addressed in Latin America to better understand, diagnose, and manage AD. A panel of AD experts noted that one of the main ways to address these is to have more funding for research on these topics [17]. The impact of certain demographic factors on disease severity needs further exploration, for one study using data from Brazil, Colombia, Mexico, and Peru found that individuals with darker skin color had lower odds of reporting good health, even after adjusting for self-reported race/ethnicity or interviewer-described race/ethnicity [35]. Public health policy regarding AD needs to be improved to address the disease burden on health, costs, and quality of life. Furthermore, inequities in access to specialized care and AD treatments need to be acknowledged and better studied within the region [31]. Some suggestions provided to improve these issues include continuing engagement in AD patient advocacy groups to increase awareness, creating a Latin American AD disease registry, and providing AD education to primary care physicians, patients, family, and caregivers [17]. One method of improving patient education and treatment compliance may include the use of community health workers (CHW). In the United States, Spanish-speaking community advisors have successfully improved health education and disease prevention among the Latin American population [38]. Regarding AD, a study evaluating a CHW-led educational program for Latin American caregivers of pediatric patients with AD reported increased adherence to emollients over a 12-week period in the CHW group compared to the control group. There was also a statistically significant increase in AD knowledge in the group exposed to the educational program, further supporting the efficacy of this intervention [39]. Figure 3 illustrates the explored topics in this review and the major issues related in LA.

## 6. Conclusions

Atopic dermatitis is a prevalent condition in Latin America that can have a substantial impact on quality of life. Diagnosing AD is challenging due to broad clinical features and lack of universal diagnostic criteria. Furthermore, lack of physician training, barriers to access, and socioeconomic inequalities hinder effective disease management. Ethnoracial disparities in AD need to be addressed, as they may impact not only in the diagnosis, but also in severity scores which are relevant parameters for evaluating the efficacy of therapeutic agents. More research needs to be performed to better understand the complexities of AD in Latin America, and special attention should be placed on patient advocacy, education, and public policy to enhance AD knowledge and improve the quality of life in these patients.

## Figures and Tables

**Table 1:** Main findings of the articles included in the study.

Authors	Year	Main Findings
Asher et al. [1]	2006	Increase in the world prevalence of eczema in children aged 6–7 and 13–14
Barreto et al. [4]	2012	LA is one of the world’s most unequal regions, with significant variation in poverty among countries
Solé et al. [5]	2010	The mean prevalence of current eczema in schoolchildren in LA was 11.3%, and prevalence varied significantly between centers in the region
Silverberg et al. [6]	2021	The global 12-month prevalence of diagnosed atopic dermatitis in the pediatric population ranged from 2.7% to 20.1%, with Brazil having the highest prevalence.
García et al. [7]	2020	A total of 42.8% of children aged 1–6 years old in Bogotá, Colombia presented with a dermatologic disease
		AD prevalence in this cohort was 6.5%
Solé et al. [8]	2006	Mean prevalence rates of atopic AD in 20 Brazilian cities was 8.2% among children ages 6–7 and 5.0% among adolescents aged 13–14
Miot et al. [9]	2023	One-year prevalence of AD in Brazil population-based telephone survey: age-adjusted prevalence of AD of 2.27%
Arruda et al. [10]	2021	Common disease manifestations in adults with AD were pruritus and erythema (Brazil)
		A total of 83% of patients had moderate to severe AD, with lichenified/eczematous pattern
		Most common topical treatments: corticosteroids and emollients
		Systemic treatments frequently reported: antihistamines and oral corticosteroids
Cárdernas et al. [12]	2022	Chilean patients: 11.1% of AD patients: carriers of filaggrin loss-of-function variants versus 5.2% of those without AD
		Similar prevalence of filaggrin variant carriers to that of European populations
Callou et al. [13]	2022	Patients with AD: increased expression of filaggrin and claudin-1 in the ocular surface when compared to healthy controls, despite decreased expression of these proteins in the skin
		Possible reactive response of the ocular surface to AD-related inflammation
Weiland et al. [14]	2004	Prevalence of childhood eczema symptoms correlated positively with latitude and negatively with mean annual outdoor temperature: does climate have an effect on eczema prevalence?
Orfali et al. [15]	2018	In adults with AD, staphylococcal enterotoxins led to dysfunctional CD4+ T-cells that produce IL-22
Orfali et al. [16]	2019	In patients with AD, staphylococcal enterotoxin A upregulated anergy-related genes and led to a compromised response in CD4+ T cells in response to antigen stimulation
Borzutzky et al. [17]	2022	There are many knowledge gaps in AD knowledge and research in Latin America, including updated prevalence, phenotypes and endotypes, specialist availability and distribution, and public health policy
Mesquita et al. [18]	2019	AD prevalence estimated to be at 7% in Brazil, with the majority of experts (82%) using the Hanifin and Rajka diagnostic criteria
Sanchez et al. [19]	2021	Validated clinical assessment tools should be used in AD to measure impact of treatment on disease and effects of disease on quality of life; limited use LA
		Barriers to AD treatment in LA: lack of physician and patient education, limited access to care, and lack of national clinical practice guidelines and AD assessment tools
Caraballo et al. [20]	2016	Exposure to mite allergens, helminth infections, and insects in tropical regions may affect the natural course of allergic diseases such as AD
Orfali et al. [21]	2013	Adult patients with AD: over half had moderate to severe disease, and 56% of patients with >1 hospitalization
		A total of 71/80 patients had concomitant respiratory disease
		Disease severity: positive correlation with high IgE serum levels and eosinophil count
Sanchez et al. [22]	2014	LA Society of Allergy Asthma and Immunology: strongly recommends testing for aeroallergens and food allergens in certain patients with AD
		Moisturizers, topical steroids, oral steroids, phototherapy, and cyclosporine for certain patients with AD are recommended
Alcântara-Neves et al. [23]	2014	Poor urban area in LA: 50% of children infected with at least 1 parasite Eosinophilia > 4% in 74.3% of children, total IgE > 200 IU/mL in 59.7% of children
Echeverría et al. [24]	2022	Argentinian patients: AD with significant impacts on quality of life, including mood alterations, sleep alterations, routine alterations, pain and economic impacts
Sanclemente et al. [25]	2021	AD mostly flexural, combined with either eyelid dermatitis, hand eczema, or cheilitis
		AD associated with comorbidities such as sleep disturbances, anxiety, and depression in Colombian patients
Jardim Criado et al. [26]	2023	Severe pruritus in 54.4% of patients
		Impact on quality of life among 50% of patients older than 16
Castro et al. [27]	2014	A total of 18/31 patients with AD had moderate to severe depressive symptoms, and 7/31 had severe symptoms
		A total of 96.8% of patients with AD reported stress
		A total of 45.2% of patients had significant quality of life impairments
Urrutia-Pereira et al. [28]	2017	Children with AD had higher scores on the Children Sleep Habits Questionnaire than controls, and this score significantly correlated with bedtime resistance, sleep anxiety, nighttime awakening, and daytime sleepiness
Sanchez et al. [29]	2017	In a tropical population cohort of patients with AD, following international AD guidelines and recommendations led to improvements in AD severity and quality of life
Aoki et al. [30]	2019	Brazilian consensus:
		Therapeutic basic strategy for AD includes adequate skin hydration, topical anti-inflammatory agents, avoidance of triggers, and educational programs
		Systemic immunosuppressants recommended for patients with severe refractory AD
		Patients may need to be hospitalized for control of AD flares
Sanchez et al. [31]	2023	Factors that contribute to healthcare disparities in AD in LA: lack of disease knowledge and education, unequal distribution of resources, absence of clinical practice guidelines, and cultural and linguistic barriers
Samorano et al. [32]	2021	Methotrexate for 24 weeks significantly reduced EASI, SCORAD, and pruritus in adult patients with AD and is an effective second-line therapy for moderate to severe AD
Aguiar Júnior et al. [33]	2011	A total of 45/85 children used an alternative or complementary medicine to treat their AD; Phytotherapy: most common choice
Adhikari et al. [34]	2016	Admixture impacts the genetic makeup of LA population
		Genetic and social factors play a role in the structure of the population in Latin America, with influence in biological diversity and disease susceptibility
Perreira et al. [35]	2014	In LA, patients with darker skin tones reported poorer health
		Exposure to low socioeconomic status responsible for the association between skin color and health
Batista et al. [36]	2015	Decreased expression of filaggrin and claudin-1 in lesional skin of AD patients compared to controls
		IL-17 expression and levels of Th1 and Th17 inflammatory cytokines were increased in AD patients
Barbarot et al. [37]	2022	EPI-CARE study: Pediatric AD severity correlated with higher scores on the Dermatitis Family Impact questionnaire, indicating a more severe caregiver burden, across regions
Rhodes et al. [38]	2007	The use of lay health advisors to address healthcare disparities among LA communities may be effective, but stronger data are needed to support these interventions
Chen et al. [39]	2023	Education provided by Spanish-speaking community health care workers led to an increased adherence to emollients in Hispanic children with AD

LA: Latin America; AD: atopic dermatitis.

## Data Availability

No new data were created or analyzed in this study. Data sharing is not applicable to this article.

## References

[1] Asher M.I., Montefort S., Bjorksten B., Lai C.K., Strachan D.P., Weiland S.K., Williams H., Group I.P.T.S. (2006). Worldwide time trends in the prevalence of symptoms of asthma, allergic rhinoconjunctivitis, and eczema in childhood: ISAAC Phases One and Three repeat multicountry cross-sectional surveys. Lancet.

[2] Yon J.-A.-L., Lee S.-K., Keng J.-W., Chow S.-C., Liew K.-B., Teo S.-S., Shaik Mossadeq W.M., Marriott P.J., Akowuah G.A., Ming L.C. (2022). *Cassia alata* (Linnaeus) Roxburgh for Skin: Natural Remedies for Atopic Dermatitis in Asia and Their Pharmacological Activities. Cosmetics.

[3] Beattie P.E., Lewis-Jones M.S. (2006). A comparative study of impairment of quality of life in children with skin disease and children with other chronic childhood diseases. Br. J. Dermatol..

[4] Barreto S.M., Miranda J.J., Figueroa J.P., Schmidt M.I., Munoz S., Kuri-Morales P.P., Silva J.B. (2012). Epidemiology in Latin America and the Caribbean: Current situation and challenges. Int. J. Epidemiol..

[5] Sole D., Mallol J., Wandalsen G.F., Aguirre V., Latin American I.P.S.G. (2010). Prevalence of symptoms of eczema in Latin America: Results of the International Study of Asthma and Allergies in Childhood (ISAAC) Phase 3. J. Investig. Allergol. Clin. Immunol..

[6] Silverberg J.I., Barbarot S., Gadkari A., Simpson E.L., Weidinger S., Mina-Osorio P., Rossi A.B., Brignoli L., Saba G., Guillemin I. (2021). Atopic dermatitis in the pediatric population: A cross-sectional, international epidemiologic study. Ann. Allergy Asthma Immunol..

[7] Garcia E., Halpert E., Borrero E., Ibanez M., Chaparro P., Molina J., Torres M. (2020). Prevalence of skin diseases in children 1 to 6 years old in the city of Bogota, Colombia. World Allergy Organ. J..

[8] Sole D., Wandalsen G.F., Camelo-Nunes I.C., Naspitz C.K., Group I.B. (2006). Prevalence of symptoms of asthma, rhinitis, and atopic eczema among Brazilian children and adolescents identified by the International Study of Asthma and Allergies in Childhood (ISAAC)—Phase 3. J. Pediatr. Rio J..

[9] Miot H.A., Aoki V., Orfali R.L., Sole D., Mallozi M.C., Rodrigues T.C., Silverberg J.I. (2023). The (one-year) prevalence of atopic dermatitis in Brazil: A population-based telephone survey. J. Eur. Acad. Dermatol. Venereol..

[10] Arruda L.K., Yang A.C., Aoki V., Criado R.F., Pires M.C., Lupi O., Fabricio L.H., Richman D., Silvi S. (2021). Clinical Features and Disease Management in Adult Patients with Atopic Dermatitis Receiving Care at Reference Hospitals in Brazil: The ADAPT Study. J. Investig. Allergol. Clin. Immunol..

[11] Kaufman B.P., Guttman-Yassky E., Alexis A.F. (2018). Atopic dermatitis in diverse racial and ethnic groups-Variations in epidemiology, genetics, clinical presentation and treatment. Exp. Dermatol..

[12] Cardenas G.V., Iturriaga C., Hernandez C.D., Tejos-Bravo M., Perez-Mateluna G., Cabalin C., Urzua M., Venegas-Salas L.F., Fraga J.P., Rebolledo B. (2022). Prevalence of filaggrin loss-of-function variants in Chilean population with and without atopic dermatitis. Int. J. Dermatol..

[13] Callou T.M.P., Orfali R.L., Sotto M.N., Pereira N.V., Zaniboni M.C., Aoki V., Brito M.P., Matsuda M., Santo R.M. (2022). Increased expression of Filaggrin and Claudin-1 in the ocular surface of patients with atopic dermatitis. J. Eur. Acad. Dermatol. Venereol..

[14] Weiland S.K., Husing A., Strachan D.P., Rzehak P., Pearce N., Group I.P.O.S. (2004). Climate and the prevalence of symptoms of asthma, allergic rhinitis, and atopic eczema in children. Occup. Environ. Med..

[15] Orfali R.L., da Silva Oliveira L.M., de Lima J.F., de Carvalho G.C., Ramos Y.A.L., Pereira N.Z., Pereira N.V., Zaniboni M.C., Sotto M.N., da Silva Duarte A.J. (2018). Staphylococcus aureus enterotoxins modulate IL-22-secreting cells in adults with atopic dermatitis. Sci. Rep..

[16] Orfali R.L., Yoshikawa F.S.Y., Oliveira L., Pereira N.Z., de Lima J.F., Ramos Y.A.L., Duarte A., Sato M.N., Aoki V. (2019). Staphylococcal enterotoxins modulate the effector CD4(+) T cell response by reshaping the gene expression profile in adults with atopic dermatitis. Sci. Rep..

[17] Borzutzky A., Larco J.I., Luna P.C., McElwee E., Pires M.C., Rico Restrepo M., Saez-de-Ocariz M., Sanchez J. (2022). Atopic Dermatitis in Latin America: A Roadmap to Address Data Collection, Knowledge Gaps, and Challenges. Dermatitis.

[18] Mesquita K., Colombini M., Duarte G., Ferreira S.B., Yang A., Mallozi M., Lupi O., Guidacci M., Abreu D., Paiva H. (2019). Unveiling atopic dermatitis burden in Brazil: A report from clinical assistance perspective. JBES Braz. J. Health Econ./J. Bras. De Econ. Da Saúde.

[19] Sanchez J., Cherrez-Ojeda I., Galvan C., Garcia E., Hernandez-Mantilla N., Londono Garcia A., McElwee E., Rico Restrepo M., Rivas E., Hidalgo B. (2021). The Unmet Needs in Atopic Dermatitis Control in Latin America: A Multidisciplinary Expert Perspective. Dermatol. Ther..

[20] Caraballo L., Zakzuk J., Lee B.W., Acevedo N., Soh J.Y., Sanchez-Borges M., Hossny E., Garcia E., Rosario N., Ansotegui I. (2016). Particularities of allergy in the Tropics. World Allergy Organ. J..

[21] Orfali R.L., Shimizua M.M., Takaoka R., Zaniboni M.C., Ishizaki A.S., Costa A.A., Tiba A.P.L., Sato M.N., Aoki V. (2013). Atopic dermatitis in adults: Clinical and epidemiological considerations. Rev. Da Assoc. Med. Bras..

[22] Sanchez J., Paez B., Macias A., Olmos C., de Falco A. (2014). Atopic dermatitis guideline. Position paper from the Latin American Society of Allergy, Asthma and Immunology. Rev. Alerg. Mex..

[23] Alcantara-Neves N.M., de SG Britto G., Veiga R.V., Figueiredo C.A., Fiaccone R.L., da Conceicao J.S., Cruz A.A., Rodrigues L.C., Cooper P.J., Pontes-de-Carvalho L.C. (2014). Effects of helminth co-infections on atopy, asthma and cytokine production in children living in a poor urban area in Latin America. BMC Res. Notes.

[24] Echeverria C., Angles M.V., Larralde M., Luna P.C., Mazzuoccolo L.D., Moreno P. (2022). Impact of atopic dermatitis on quality of life: A large web-based survey from Argentina. Rev. Fac. Cien Med. Univ. Nac. Cordoba.

[25] Sanclemente G., Hernandez N., Chaparro D., Tamayo L., Lopez A., Colombian Atopic Dermatitis Research G. (2021). Epidemiologic features and burden of atopic dermatitis in adolescent and adult patients: A cross-sectional multicenter study. World Allergy Organ. J..

[26] Jardim Criado R.F., Rodrigues T., de Campos L., Cestari T., Maspero J., Luna P.C., Angles M.V., Antila M. (2023). 347 The real-world burden of atopic dermatitis: MEASURE-AD multicountry study results from Brazil, Mexico and Argentina. Br. J. Dermatol..

[27] Castro C.R., Andrade M.E.B., Pires R.M.G., Pires M.C. (2021). Evaluation of depression, stress and quality of life indexes in patients with atopic dermatitis. An. Bras. Dermatol..

[28] Urrutia-Pereira M., Sole D., Rosario N.A., Neto H.J.C., Acosta V., Almendarez C.F., Avalos M.M., Badellino H., Berroa F., Alvarez-Castello M. (2017). Sleep-related disorders in Latin-American children with atopic dermatitis: A case control study. Allergol. Immunopathol..

[29] Sanchez J., Toro Y., Cardona R. (2017). Clinical impact in the real life of guidelines recommendations for atopic dermatitis in a tropical population (TECCEMA cohort). Rev. Alerg. Mex..

[30] Aoki V., Lorenzini D., Orfali R.L., Zaniboni M.C., Oliveira Z.N.P., Rivitti-Machado M.C., Takaoka R., Weber M.B., Cestari T., Gontijo B. (2019). Consensus on the therapeutic management of atopic dermatitis—Brazilian Society of Dermatology. An. Bras. Dermatol..

[31] Sanchez J., Ale I.S., Angles M.V., Fogelbach G.G., Jansen A.M., Takaoka R., Borzutzky A. (2023). Healthcare Disparities in Atopic Dermatitis in Latin America: A Narrative Review. Dermatol. Ther..

[32] Samorano L.P., Takaoka R., Zaniboni M.C., Aoki V. (2021). Methotrexate for atopic dermatitis in adults: A prospective study from a reference center in Brazil. J. Dtsch. Dermatol. Ges..

[33] Aguiar Junior Ndos R., Costa I.M. (2011). The use of alternative or complementary medicine for children with atopic dermatitis. An. Bras. Dermatol..

[34] Adhikari K., Mendoza-Revilla J., Chacon-Duque J.C., Fuentes-Guajardo M., Ruiz-Linares A. (2016). Admixture in Latin America. Curr. Opin. Genet. Dev..

[35] Perreira K.M., Telles E.E. (2014). The color of health: Skin color, ethnoracial classification, and discrimination in the health of Latin Americans. Soc. Sci. Med..

[36] Batista D.I., Perez L., Orfali R.L., Zaniboni M.C., Samorano L.P., Pereira N.V., Sotto M.N., Ishizaki A.S., Oliveira L.M., Sato M.N. (2015). Profile of skin barrier proteins (filaggrin, claudins 1 and 4) and Th1/Th2/Th17 cytokines in adults with atopic dermatitis. J. Eur. Acad. Dermatol. Venereol..

[37] Barbarot S., Silverberg J.I., Gadkari A., Simpson E.L., Weidinger S., Mina-Osorio P., Rossi A.B., Brignoli L., Mnif T., Guillemin I. (2022). The Family Impact of Atopic Dermatitis in the Pediatric Population: Results from an International Cross-sectional Study. J. Pediatr..

[38] Rhodes S.D., Foley K.L., Zometa C.S., Bloom F.R. (2007). Lay health advisor interventions among Hispanics/Latinos—A qualitative systematic review. Am. J. Prev. Med..

[39] Chen H.W., Limmer E.E., Joseph A.K., Kinser K., Trevino A., Valencia A., Weinheimer R.A., Youssef S.H., Cervantes C., Guzman M.T. (2023). Efficacy of a lay community health worker (promotoras de salud) program to improve adherence to emollients in Spanish-speaking Latin American pediatric patients in the United States with atopic dermatitis: A randomized, controlled, evaluator-blinded study. Pediatr. Dermatol..

[40] Brunner P.M., Guttman-Yassky E. (2019). Racial differences in atopic dermatitis. Ann. Allergy Asthma Immunol..

[41] Noda S., Suarez-Farinas M., Ungar B., Kim S.J., de Guzman Strong C., Xu H., Peng X., Estrada Y.D., Nakajima S., Honda T. (2015). The Asian atopic dermatitis phenotype combines features of atopic dermatitis and psoriasis with increased TH17 polarization. J. Allergy Clin. Immunol..

[42] Hanifin J.M., Rajka G. (1980). Diagnostic Features of Atopic-Dermatitis. Acta Derm.-Venereol..

[43] Hanifin J.M., Baghoomian W., Grinich E., Leshem Y.A., Jacobson M., Simpson E.L. (2022). The Eczema Area and Severity Index-A Practical Guide. Dermatitis.

[44] Simpson E., Bissonnette R., Eichenfield L.F., Guttman-Yassky E., King B., Silverberg J.I., Beck L.A., Bieber T., Reich K., Kabashima K. (2020). The Validated Investigator Global Assessment for Atopic Dermatitis (vIGA-AD): The development and reliability testing of a novel clinical outcome measurement instrument for the severity of atopic dermatitis. J. Am. Acad. Dermatol..

[45] Simpson E.L., Bissonnette R., Paller A.S., King B., Silverberg J.I., Reich K., Thyssen J.P., Doll H., Sun L., DeLozier A.M. (2022). The Validated Investigator Global Assessment for Atopic Dermatitis (vIGA-AD): A clinical outcome measure for the severity of atopic dermatitis. Br. J. Dermatol..

[46] Nomura T., Wu J., Kabashima K., Guttman-Yassky E. (2020). Endophenotypic Variations of Atopic Dermatitis by Age, Race, and Ethnicity. J. Allergy Clin. Immunol. Pract..

